# A cause for childhood ataxia

**DOI:** 10.7554/eLife.14523

**Published:** 2016-03-01

**Authors:** Joan S Steffan

**Affiliations:** Department of Psychiatry & Human Behavior, University of California Irvine, Irvine, United Statesjssteffa@uci.edu

**Keywords:** ataxia, next generation sequencing, autophagy, *D. melanogaster*, Human, *S. cerevisiae*

## Abstract

Genetic studies uncover a mutation in a widely conserved protein as the cause of a neurological disorder in two brothers.

**Related research article** Kim M, Sandford E, Gatica D, Qui Y, Liu X, Zheng Y, Schulman B, Xu J, Semple I, Ro SH, Kim B, Mavioglu RN, Tolun A, Jipa A, Takaat S, Karpati M, Li JZ, Yapici Z, Juhasz G, Lee JH, Klionsky DJ, Burmeister M. 2016. Mutation of *ATG5* reduces autophagy and leads to ataxia with developmental delay. *eLife* **5**:e012245. doi: 10.7554/eLife.12245

**Image** The 122^nd^ amino acid in the ATG5 protein appears important for its function

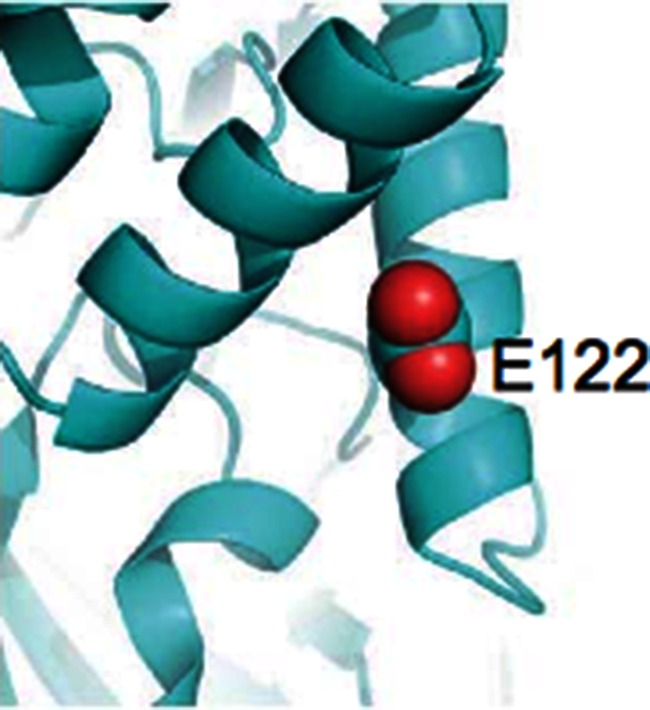

Over 10 years ago, physicians examined two Turkish brothers, aged five and seven, because they had started walking later than expected and now walked with a “drunken sailor” gait. Repeated visits to the doctor did not lead to any improvement, and it was later revealed the two boys had underdeveloped cerebellums – the part of the brain that coordinates and regulates muscular activity. The boys’ parents and two other brothers did not show symptoms, but the parents were later found to be third cousins. This discovery suggested that the loss of movement control observed in the brothers (which is more formally called ataxia) might be due to a recessive genetic mutation. In such cases, both parents carry a mutated version of a gene and a non-mutated version without obvious effect; however, it is possible that some of their children inherit only the mutated versions of the gene.

Now, in eLife, Jun Hee Lee, Daniel Klionsky, Margit Burmeister and collaborators – including Myungjin Kim and Erin Sandford of the University of Michigan as joint first authors – report the results of a search for a mutation that could explain the two brothers’ ataxia and delayed development (Kim et al., 2016). The team – who include researchers from the US, Turkey and Hungary – initially performed genetic tests on the brothers, their siblings and their mother to narrow down to a specific region of a single chromosome. Next, they looked for a mutation within this region that was found in the ataxic brothers but not the general Turkish population, in the hope of finding the cause of the disease.

Eureka! Kim, Sandford et al. found a damaging DNA mutation within the search region on both versions of the chromosome. The mutation changed the 122^nd^ amino acid of a protein called ATG5 from a glutamic acid (often simply labeled as an ‘E’) to an aspartic acid (‘D’). But can such a relatively mild change in a protein sequence have caused the ataxia?

ATG5 was first identified in yeast as a protein that is involved in a process called autophagy that breaks down materials (including proteins and organelles) within cells so that they can be recycled (Tsukada and Ohsumi, 1993; Mizushima et al., 1998). Autophagy is important because a build-up of certain molecules within cells can cause disease (Klionsky and Codogno, 2013), and many human diseases – from cancer to heart disease – are thought to involve problems with autophagy regulation (Choi et al., 2013; Katsuno et al., 2014). Blocking the production of ATG5 in the brains of mice also leads to a progressive loss of neurons (Hara et al., 2006). It is perhaps not a surprise after all that a mutation that affects ATG5 might be behind childhood ataxia.

So, what does the mutation (called E122D for short) do to ATG5? The glutamic acid that is mutated in the ataxic boys is conserved across many species from yeast to man, suggesting it is important for the activity of this protein and has therefore been unchanged during evolution (Figure 1). Kim, Sandford et al. examined the position of this conserved glutamic acid within the protein’s three-dimensional structure (Otomo et al., 2013.). They realized that this mutation might stop ATG5 from fusing with ATG12, another core autophagy protein that is required for ATG5’s activity. Kim, Sandford et al. then went on to find that autophagy was impaired in cells taken from the ataxic brothers. They also found the levels of fused ATG12 and ATG5 were reduced, and confirmed that the E122D mutation interfered with the fusion of ATG12 and ATG5 in human cells grown in the laboratory.

**Figure 1.**
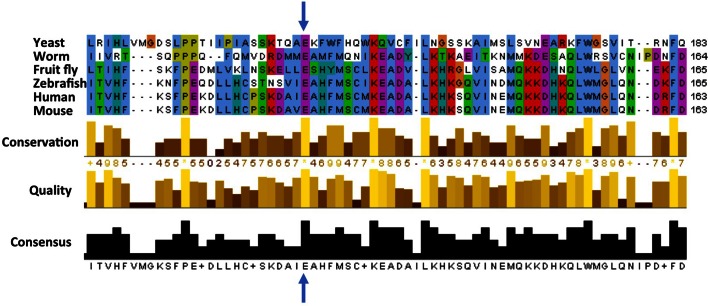
Cross-species comparison of the amino acid sequences of ATG5 proteins. Different species have very similar ATG5 proteins. For example, the glutamic acid (E, indicated by blue arrows) at position 122 in the human version of ATG5 is conserved in yeast (*Saccharomyces cerevisiae*), worms (*Caenorhabditis elegans*), fruit flies (*Drosophila melanogaster*), zebrafish (*Danio rerio*), and mice (*Mus musculus*). Only part of the sequence is shown for each protein; the amino acid number for each protein is shown on the right. Kim, Sandford et al. found that the glutamic acid at position 122 was altered to an aspartic acid (not shown) in two Turkish brothers with childhood ataxia. Sequence alignments were performed as previously described (Steffan, 2010).

Since ATG5 is found in many different species, Kim, Sandford et al. then went on to study the effect of the E122D mutation in yeast and fruit flies. Mutating the corresponding glutamic acid within the yeast protein caused a 30–50% reduction in autophagy, which was triggered by starving the yeast cells. Flies that were engineered to make the human ATG5 protein with the E122D mutation instead of their own version of ATG5 had problems with movement. This was not seen in flies that made the non-mutated form of the human protein; however, flies that did not make ATG5 at all showed even worse symptoms. These experiments support the idea that the E122D mutation within ATG5 causes a reduction, but not a complete loss, of its function as a core autophagy protein.

Kim, Sandford et al. conclude that the childhood ataxia observed in the Turkish brothers may well have been caused by a reduction in ATG5’s role in autophagy. Their work is the first to link a human disease to mutation in a gene for a core autophagy protein, and demonstrates the fundamental importance of autophagy in brain health.
